# Supplementary material for: Dynamic global acetylation remodeling during the yeast heat shock response
Source: bioRxiv. 2025 Jan 10:2025.01.10.632339. Preprint. [Version 1] doi: 10.1101/2025.01.10.632339 (PMC11812598; doi:10.1101/2025.01.10.632339)
Supplement: Supplement 5 [file NIHPP2025.01.10.632339v1-supplement-5.pdf]

bioRxiv preprint doi: https://doi.org/10.1101/2025.01.10.632339; this version posted January 10, 2025. The copyright holder for this preprint (which was not certified by peer review) is the author/funder, who has granted bioRxiv a license to display the preprint in perpetuity. It is made available under aCC-BY 4.0 International license.

Supplemental Figures

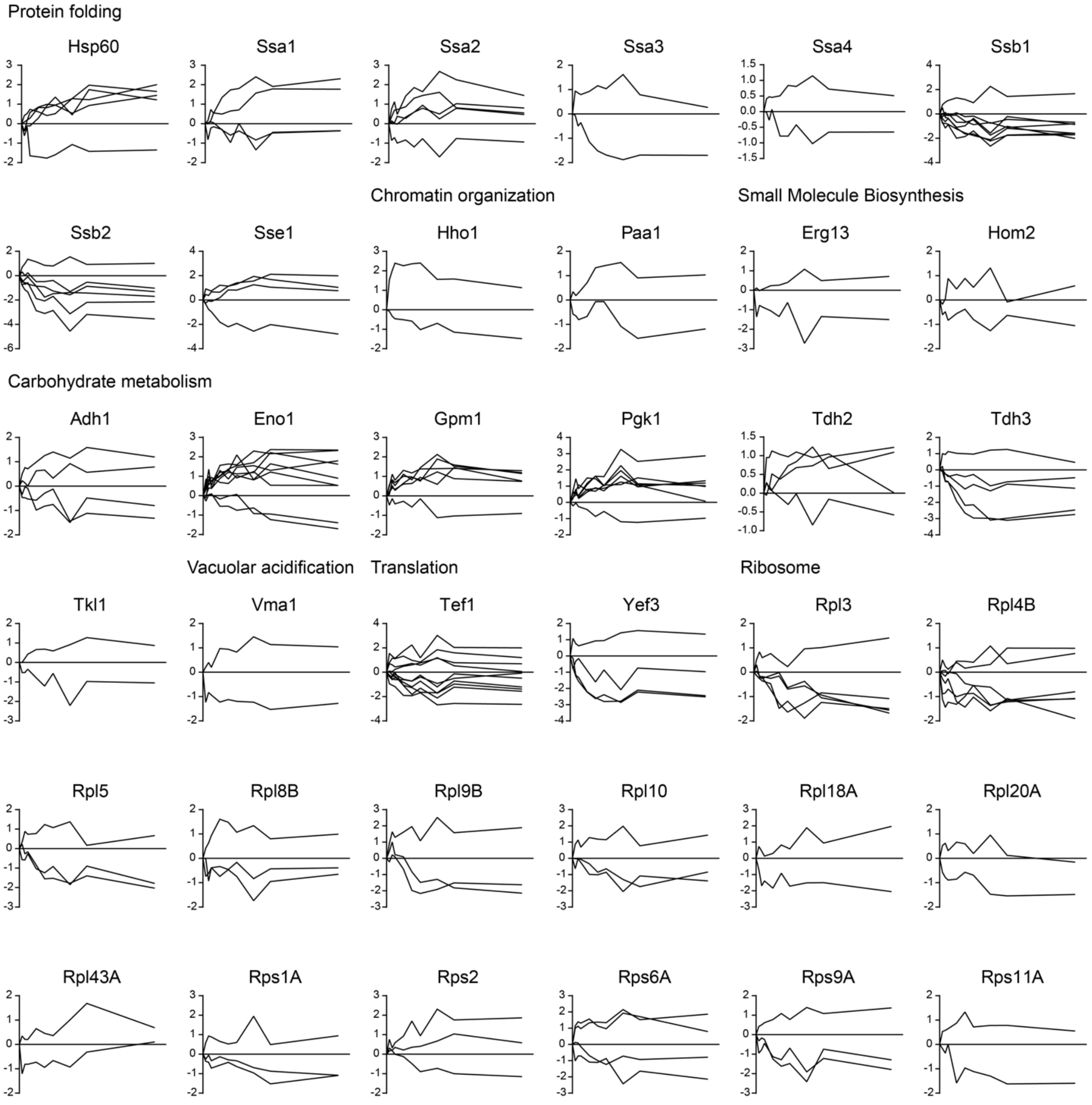

Figure S1. 36 proteins experience both increasing and decreasing acetyl residues. For each protein, the line represents the average log_2 fold change (y-axis) of one residue over the 240-min 25°C to 37°C time-course (x-axis). Proteins are grouped based on their annotated functions.

bioRxiv preprint doi: https://doi.org/10.1101/2025.01.10.632339; this version posted January 10, 2025. The copyright holder for this preprint (which was not certified by peer review) is the author/funder, who has granted bioRxiv a license to display the preprint in perpetuity. It is made available under aCC-BY 4.0 International license.

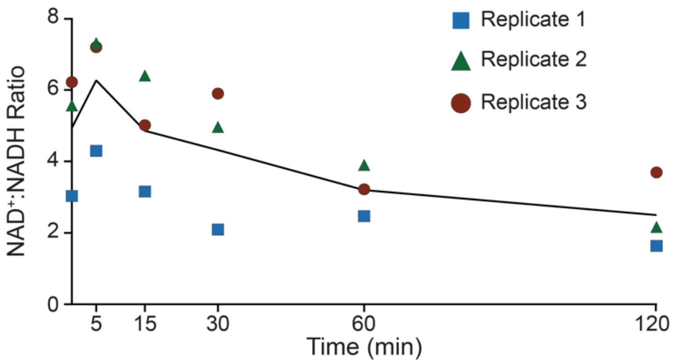

Figure S2. The NAD$^+$:NADH ratio initially spikes and then gradually decreases during heat shock. The NAD$^+$:NADH ratio was monitored across a 120 min heat shock (25°C to 37°C) via a luminescence assay.

bioRxiv preprint doi: https://doi.org/10.1101/2025.01.10.632339; this version posted January 10, 2025. The copyright holder for this preprint (which was not certified by peer review) is the author/funder, who has granted bioRxiv a license to display the preprint in perpetuity. It is made available under aCC-BY 4.0 International license.

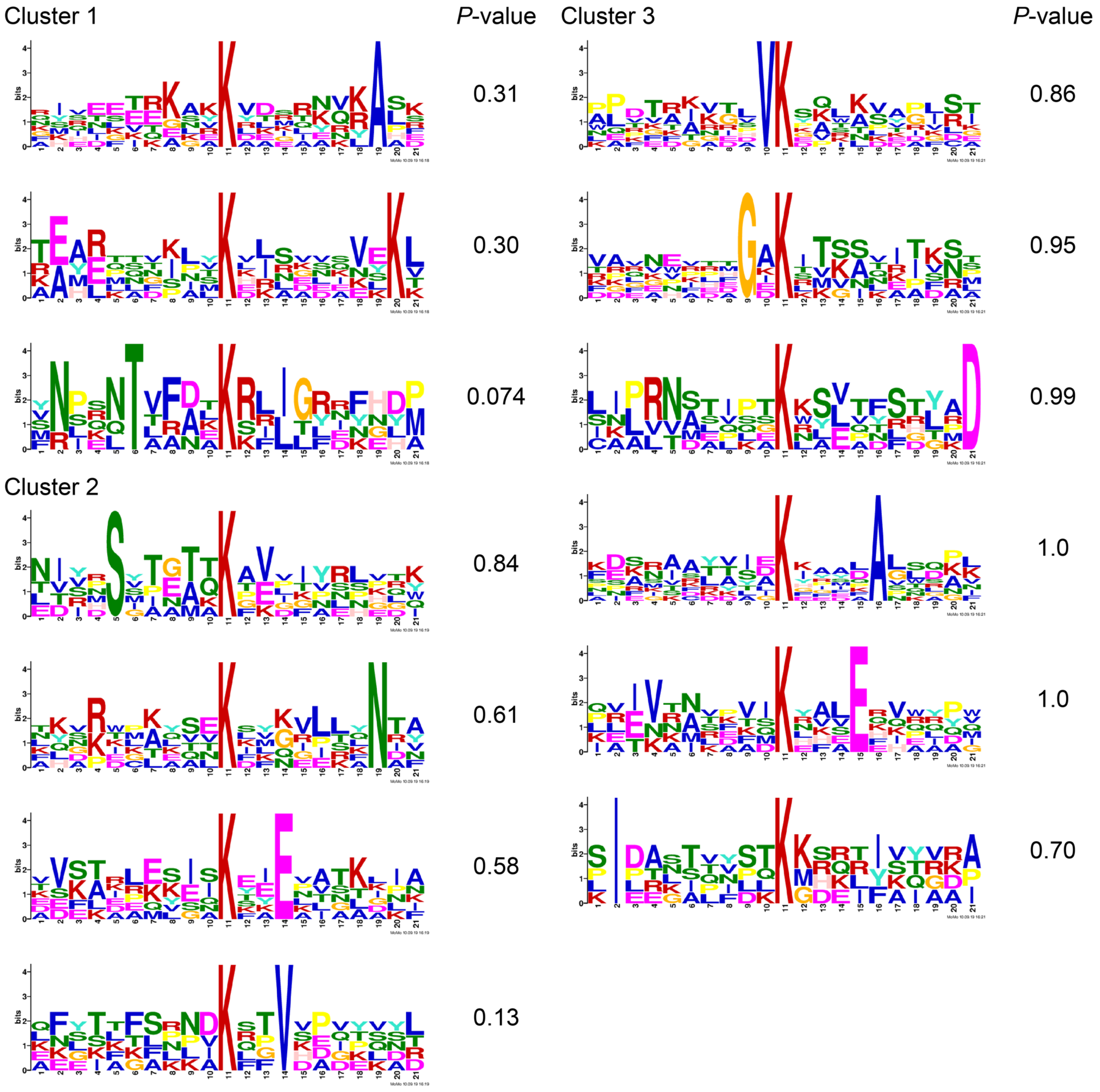

Figure S3. Motif analysis of clusters with increasing acetylation. Motif analysis of Clusters 1-3 from Figure 4A was performed using MoMo.

`

bioRxiv preprint doi: https://doi.org/10.1101/2025.01.10.632339; this version posted January 10, 2025. The copyright holder for this preprint (which was not certified by peer review) is the author/funder, who has granted bioRxiv a license to display the preprint in perpetuity. It is made available under aCC-BY 4.0 International license.

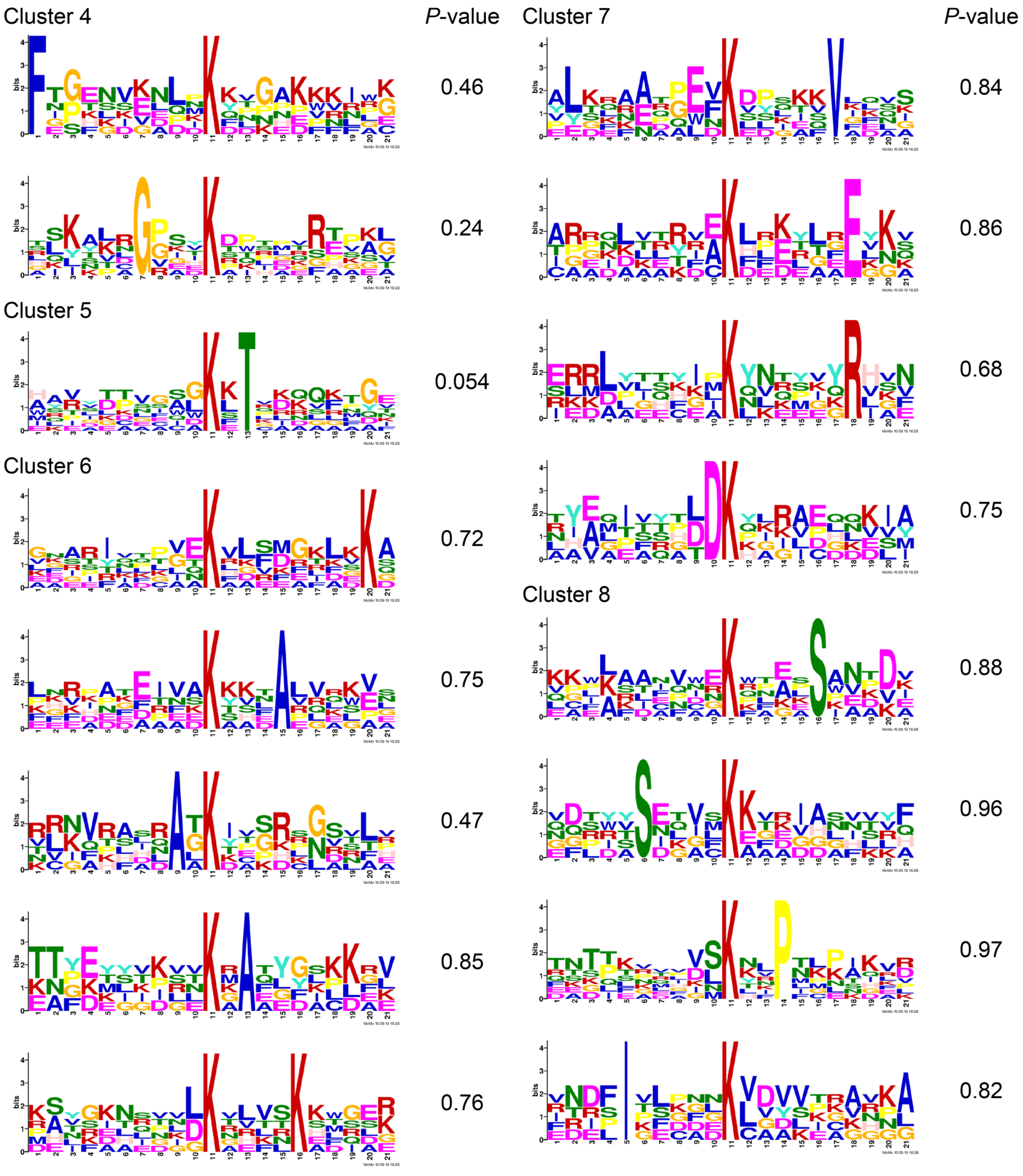

Figure S4. Motif analysis of clusters with decreasing acetylation. Motif analysis of Clusters 4-8 from Figure 4A was performed using MoMo.

bioRxiv preprint doi: https://doi.org/10.1101/2025.01.10.632339; this version posted January 10, 2025. The copyright holder for this preprint (which was not certified by peer review) is the author/funder, who has granted bioRxiv a license to display the preprint in perpetuity. It is made available under aCC-BY 4.0 International license.

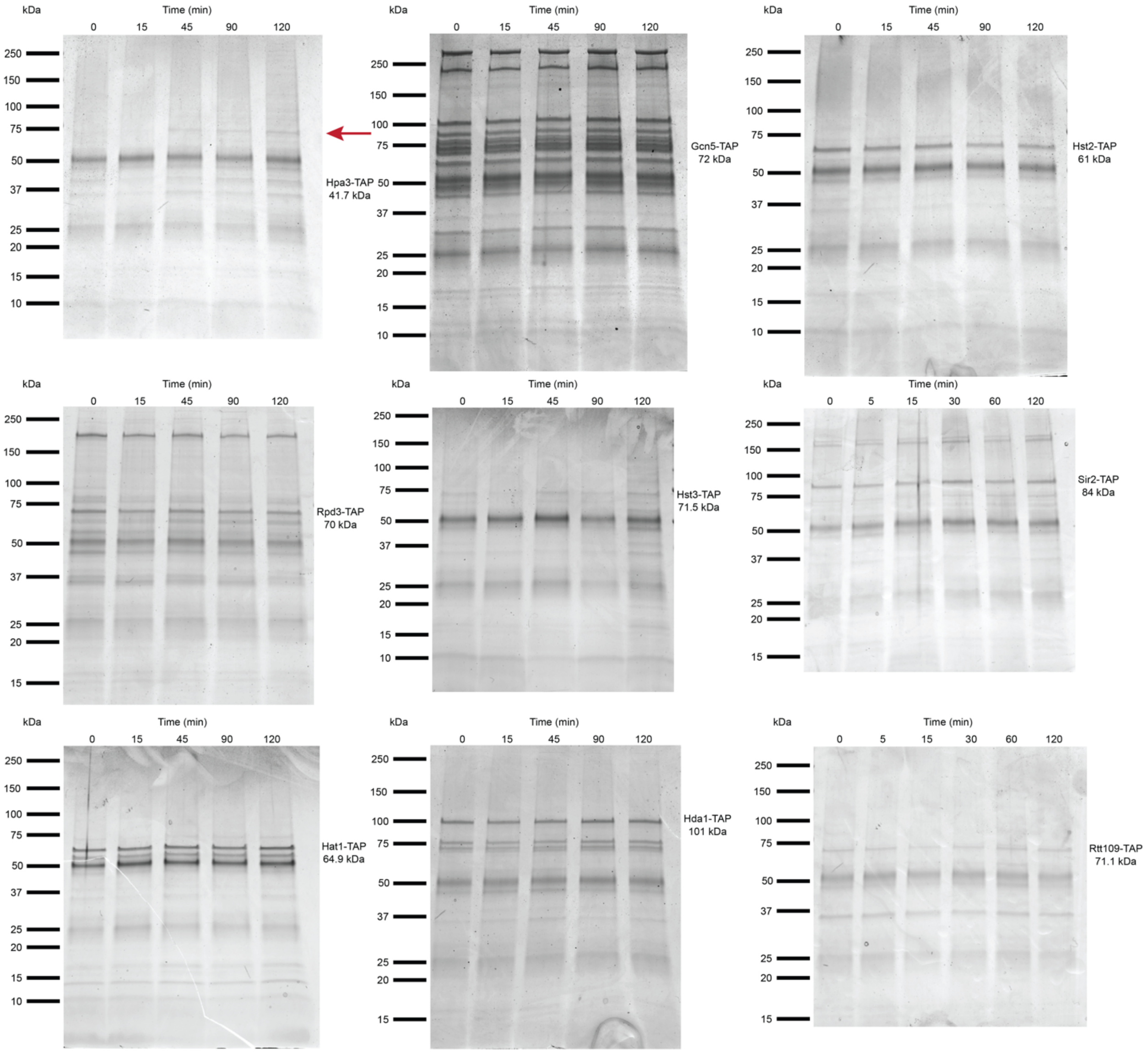

Figure S5. Tap co-immunoprecipitations of KAT and KDAC enzymes and interacting proteins. Identified KAT and KDAC enzymes and interacting proteins were co-immunoprecipitated and visualized with blue-silver staining. One interacting protein changed in abundance for the Hpa3 co-immunoprecipitation (red arrow), but could not be identified. No changes were observed for the other KATs and KDACs shown in the figure.

bioRxiv preprint doi: https://doi.org/10.1101/2025.01.10.632339; this version posted January 10, 2025. The copyright holder for this preprint (which was not certified by peer review) is the author/funder, who has granted bioRxiv a license to display the preprint in perpetuity. It is made available under aCC-BY 4.0 International license.

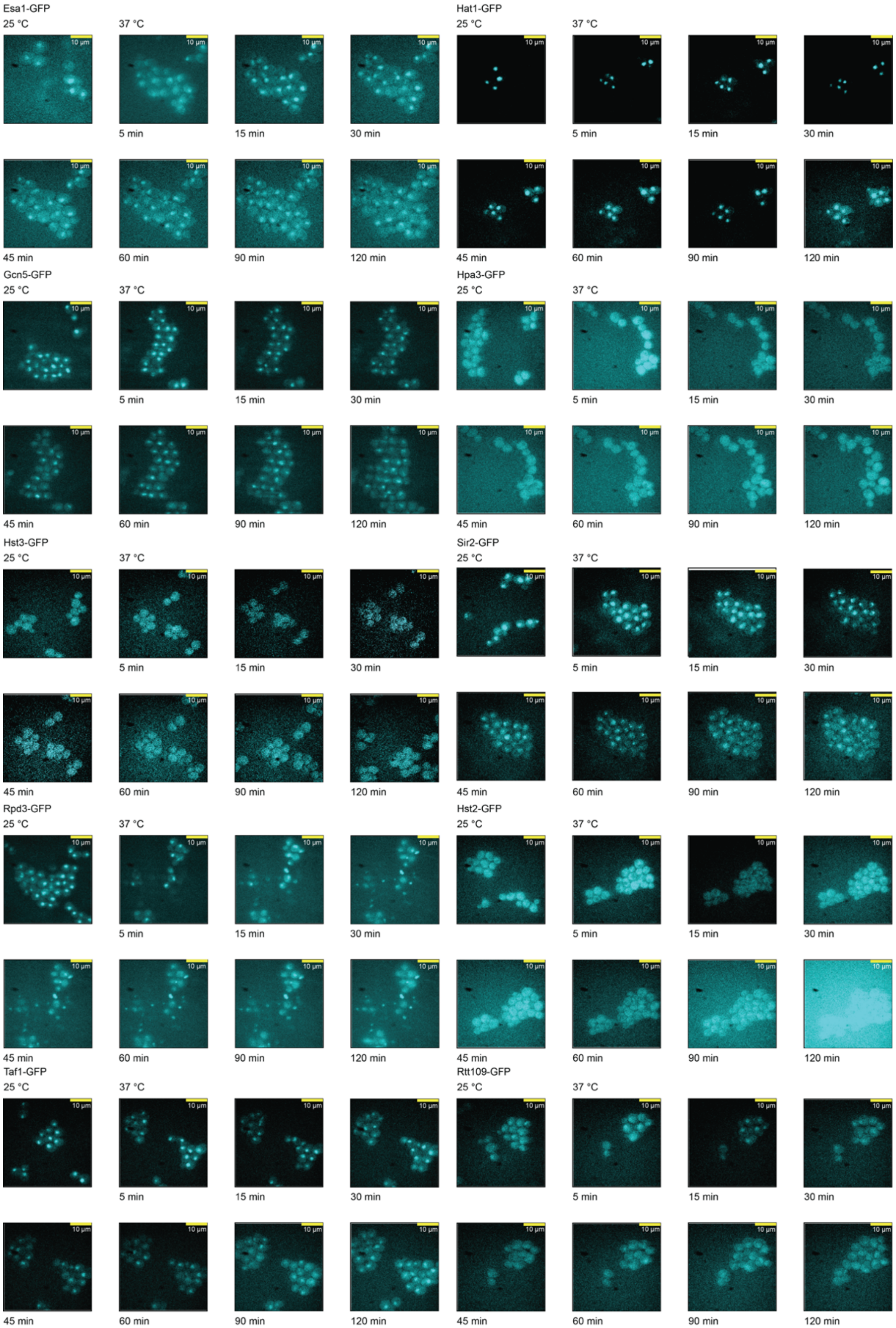

Figure S6. Fluorescence microscopy of KATs and KDACs. Strains carrying the depicted KAT or KDAC-GFP fusions were visualized at 37°C for 2 hours to determine possible changes in localization. None of the depicted proteins show changes in localization.
